# Calculation method of spherically expanding flame propagation radius to consider ignition electrode effects

**DOI:** 10.1038/s41598-024-58940-9

**Published:** 2024-04-10

**Authors:** Likang Fan, Xin Fu, Mingjie Hu, Yan Yan, Zinong Zuo, Zhiqiang Han, Jia Fang, Bang Xiao

**Affiliations:** 1 Key Laboratory of Fluid and Power Machinery of Ministry of Education, Xihua University, Chengdu, 610039 China; 2 Key Laboratory of Vehicle Measurement, Control and Safety of Sichuan Province, Xihua University, Chengdu, 610039 China; 3 Engineering Research Center of Ministry of Education for Intelligent Air-Ground Fusion Vehicles and Control, Xihua University, Chengdu, 610039 China

**Keywords:** Spherically expanding flame, Flame propagation radius, Ignition electrodes, Calculation method, Mean radius, Fossil fuels, Imaging and sensing

## Abstract

Ignition electrodes have an immense impact on the accurate measurement of the flame propagation spherical radius. In this study, a flame-radius calculation method is designed. The method is able to eliminate effects due to the ignition electrodes. The adaptability and optimization effects of the proposed method are analyzed. The results show that the ratio of the angle is affected by the ignition electrodes under the Han II method. There are three obvious divisions include a high-value area, a sharp-variation area, and a mild-variation area. The ratio of the angle affected by the ignition electrodes is only applicable to the mild-variation region when the flame presents respective convex and concave distributions. For these distributions, the increment rate of the mean radius is 0.4–0.85% and 0.42–3.19%. The reduced rate of the standard deviation of the radius extraction value is 11.91–22.1% and 5.13–17.99%, and the reduced rate of the radius extraction value range is 20.32–39.51% and 0.32–8.09%.

## Introduction

The laminar flame is the most basic combustion phenomenon that comprehensively embodies the characteristics of the gas flow property, chemical reaction, and molecular transport. It is an important means of developing and verifying the kinetic mechanism of a fuel chemical reaction^1,2^. It is also an important basis for the study of turbulent flames^3^. Moreover, it provides basic data for engine combustion simulation and optimization research^4^. To fully elucidate the laminar flame, an effective method is to obtain the flame propagation radius using a constant volume incendiary bomb and high-speed camera. Accordingly, Schlieren images are obtained, processed, and calculated. Therefore, the calculation method for the high-precision flame propagation radius is the primary input condition that is used to understand the characteristics of laminar flame combustion. Meanwhile, it is the key to determining the accuracy of laminar flame propagation velocity.

Many studies have been conducted on flame radius calculation methods. Some examples of common methods of this type are the equivalent area method^5,6^, random circle detection algorithm^7^, circle fitting^8–10^, and center measurement method^11^. Gu^12^, Milton^13^, and Zhang^14^ determines the flame radius by calculating the equivalent area. Chen et al.^6^ proposes a random circle detection algorithm to measure the flame radius by determining the true circle according to the distance threshold requirements. Broustail^8^, Han^9^, Bouvet^10^, DuttaRoy^15^, Gong^16^, and Tahtouh^17^ calculates the flame propagation radius by circle fitting of the flame front-edge points. Zuo et al.^11^ extracted the flame propagation radius through center measurement method. Wu et al.^18^uses operators to detect flame edges and improve the traditional spherical flame arc fitting method to calculate the flame radius. Li et al.^5^ applies the inter-frame difference method to process the images. They use the Random Circle Detection (RCD) algorithm, equivalent area method to identify flame rings, calculate the flame radius and center position.

**Figure 1:**
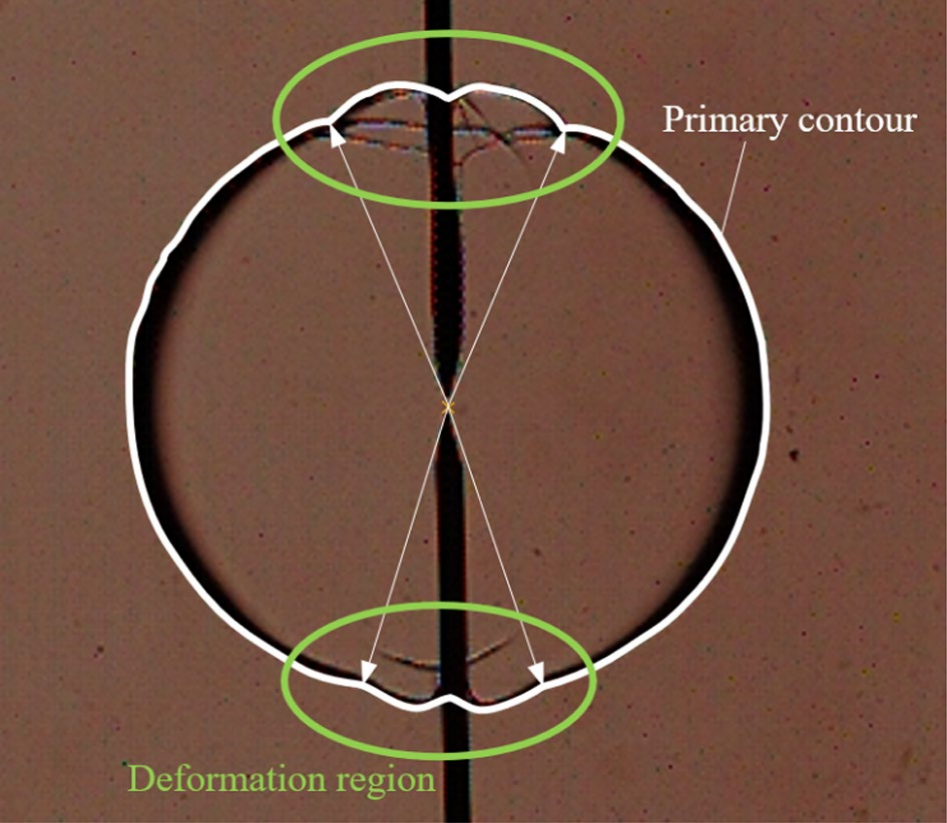
Deformation of flame profile caused by the ignition electrode.

By conducting in-depth research on the flame radius calculation method, some researchers determine that the flame radius near the ignition electrodes appeared as flame bumps and non-standard circle phenomena. Liang et al.^19^ use the Canny edge detection operator to detect the front edge of the flame in Schlieren images and then compare the difference between the arc fitting method and the flame area method in calculating the flame radius. They selected the area method for the flame radius calculation. They find out that the shape of the spherical flame is affected by electrode factors in the propagation process, which leads to a certain protrusion near the ignition electrodes. Wang et al.^20^ applies the Schlieren method to scale the recorded flame images and measure the flame radius. It is determined that the cooling effects of the ignition electrodes caused by the flame to propagate slowly along the ignition electrode direction. Han et al.^21^ confirmed that the existence of ignition electrodes is one of the main reasons for the distortion of the flame propagation radius. The existence of the ignition electrode leadS to an increase in the dispersion of the radius extraction value. The flame profile deformation caused by the ignition electrode is shown in Fig. 1.

Therefore, some scholars have gradually begun to study the phenomenon that the flame propagation radius near the ignition electrode produces a non-standard circle. Mevel and Lafosse^22^ writes a software program that takes the radius of the equivalent circle as the flame propagation radius. Although the nonstandard circle is considered in this method, the effects of ignition electrodes on accurate flame radius measurement are not separately considered. Liang^19^ and Wang^20^ only proves the effects of the ignition electrodes on the flame radius, but they do not address the effects on the accurate measurement of the flame radius. Varea et al.^23^ proposes a method of flame radius post-processing based on the effects of ignition electrodes on the flame radius. They apply this method to the extraction of the laminar combustion parameters of ethanol/air and methanol/ethanol/isooctane/air mixture. Although they propose a corresponding calculation method for the effects of ignition electrodes on the flame propagation radius, they only solve the problem of the non-standard circle of the flame surface engendered by the electrodes. The influence range of the ignition electrode is not further studied. Han et al.^21^ establishes an optimization method for the influence of ignition electrodes. However, the optimization method can only solve the problem of electrode interference with the protrusion of the flame propagation radius near the ignition electrodes in certain conditions. It is not suitable for the non-standard flame radius near most ignition electrodes.

**Figure 2:**
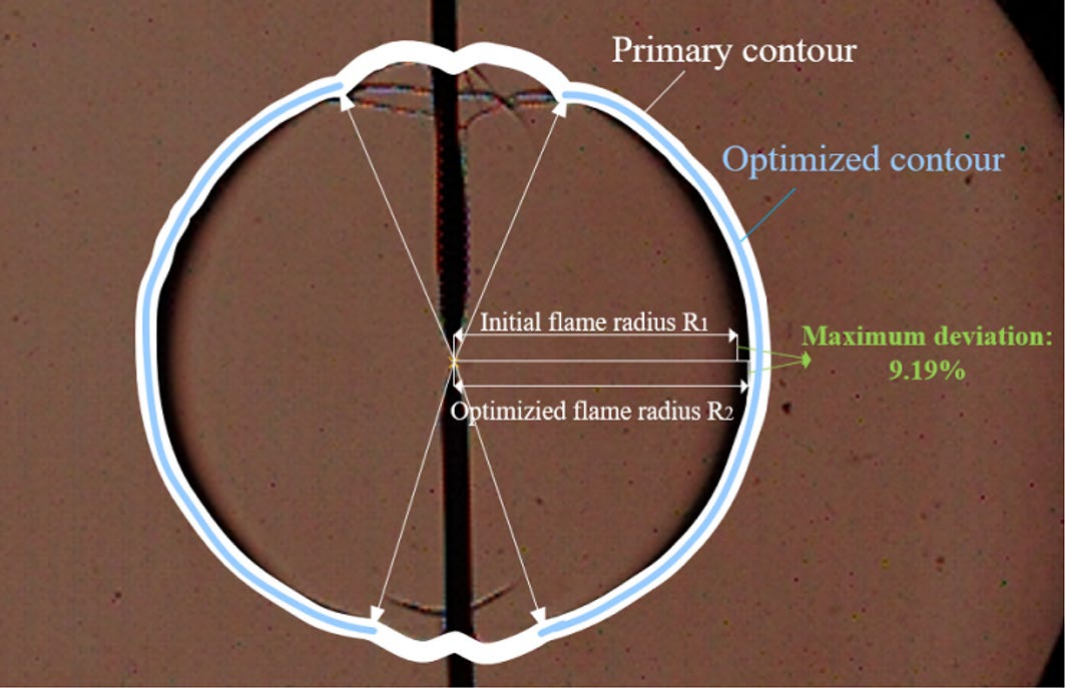
Flame profile without ignition electrode effect.

In summary, ignition electrodes that cause flame bumps and non-standard circle phenomena significantly affects the accurate measurement of the spherical radius of the flame propagation. Nevertheless, many researchers have only focused on the measurement of the laminar flame propagation radius. To date, few calculation methods have been devised that eliminate the effects of ignition electrodes on the laminar flame propagation radius, which would enable the spherical flame propagation radius to be accurately measured. Therefore, a flame radius calculation method is herein proposed to remove the effects of ignition electrodes based on the above flame radius calculation method. Figure 2 shows the flame profile without the influence of ignition electrode. Compared with the equivalent circle area method, the circle fitting method and the center measurement method, the maximum difference of the calculated flame radius is 4.54%, 5.19% and 9.17%, respectively. This method not only provides a means of supporting the accurate measurement of the spherical flame propagation radius, but also sets a foundation for scholars to conduct high-precision laminar combustion studies and contribute to the advancement of basic combustion research.

## Experimental methods and specifications

### Experimental setup

**Figure 3:**
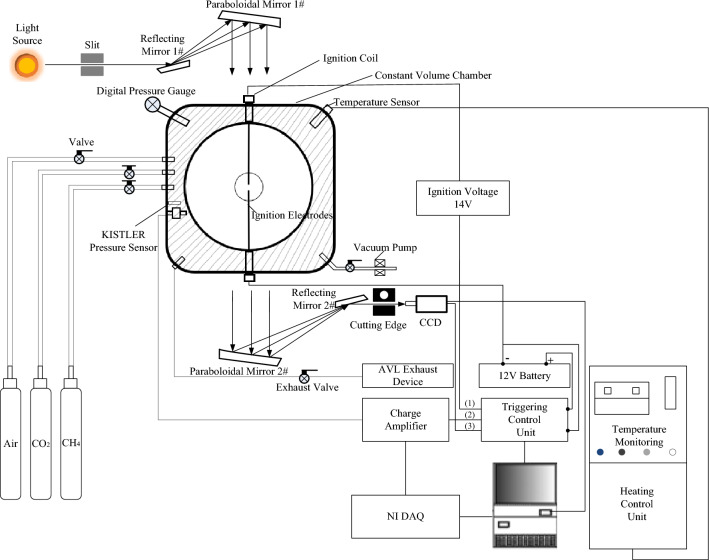
Schematic of the CVCV experimental system.

The experiments of this study are conducted in a constant volume combustion vessel (CVCV), which is a sphere with an inner diameter of 260 mm. The CVCV has two 100 mm quartz windows on each side to provide visual access for the Schlieren photography system. Electrodes extend into the center of the CVCV to realize ignition in Fig. 3. The initial temperature in the CVCV is controlled within 600 K by using a heating control system. A high-speed camera is used to record the flame propagation process at an image resolution of 512 × 512. The parameters of the sensors are used in this test are shown in Table 1. Moreover, all experimental protocols are approved by the Xihua University.

**Table 1:** Parameters of sensors.

Sensor name	Model	Specification	Accuracy	Manufacturer
Pressure sensor	6125C	0–30 MPa	± 1%	Switzerland Kistler
Charge amplifier	5018A	± (2–2,200,000)pC	FS > 10pC ± 2% FS > 100pC ± 0.6% FS ≥ 100pC ± 0.3%	Switzerland Kistler
Acquisition card	USB-6356	− 10 to 10 V	± 1% of range	America NI
K type thermocouple temperature sensor	WRNK-191	− 50 to 1100 °C	± 0.75%	Dongtai Hongtai Electric Heating Technology
Precise digital pressure gauge	OBT-200	0–1.6 MPa	0.5%	Jiangsu Obest Automation Technology

### Experimental method

Extract the residual exhaust gas from the CVCV after the previous test and bring it to a vacuum state. Then, the gas of each component is slowly entered into the CVCV by using the corresponding partial-pressure method, and the CVCV is simultaneously heated until the initial temperature is reached. Only the flame images with 6 mm < R < 25 mm are used to avoid the effects of ignition energy and combustion pressure^24^. Moreover, flame images with obvious cellular structures are omitted^25^.

The test conditions are shown in Table 2. The diluent gas fraction $({\Phi }_{r})$ represents the volume fraction of diluted gas (CO_2_) in all the gas mixtures. The diluting gas fraction is defined as the volume fraction of the diluting gas in the mixed gas, which is used to describe the relative content of the diluting gas in the mixed gas. The equivalence ratio is defined as the ratio of the theoretically required amount of air for complete combustion to the actual amount of air supplied during fuel combustion.

**Table 2:** Experimental conditions.

Condition	Initial test conditions
Condition 1	${T}_{u}=323$ K, ${P}_{u}=0.3$ MPa, $\Phi =1.0$, ${\Phi }_{r}=0\%$
Condition 2	${T}_{u}=423$ K, ${P}_{u}=0.2$ MPa, $\Phi =1.1$, ${\Phi }_{r}=16\%$

### Photographic processing procedure

**Figure 4:**
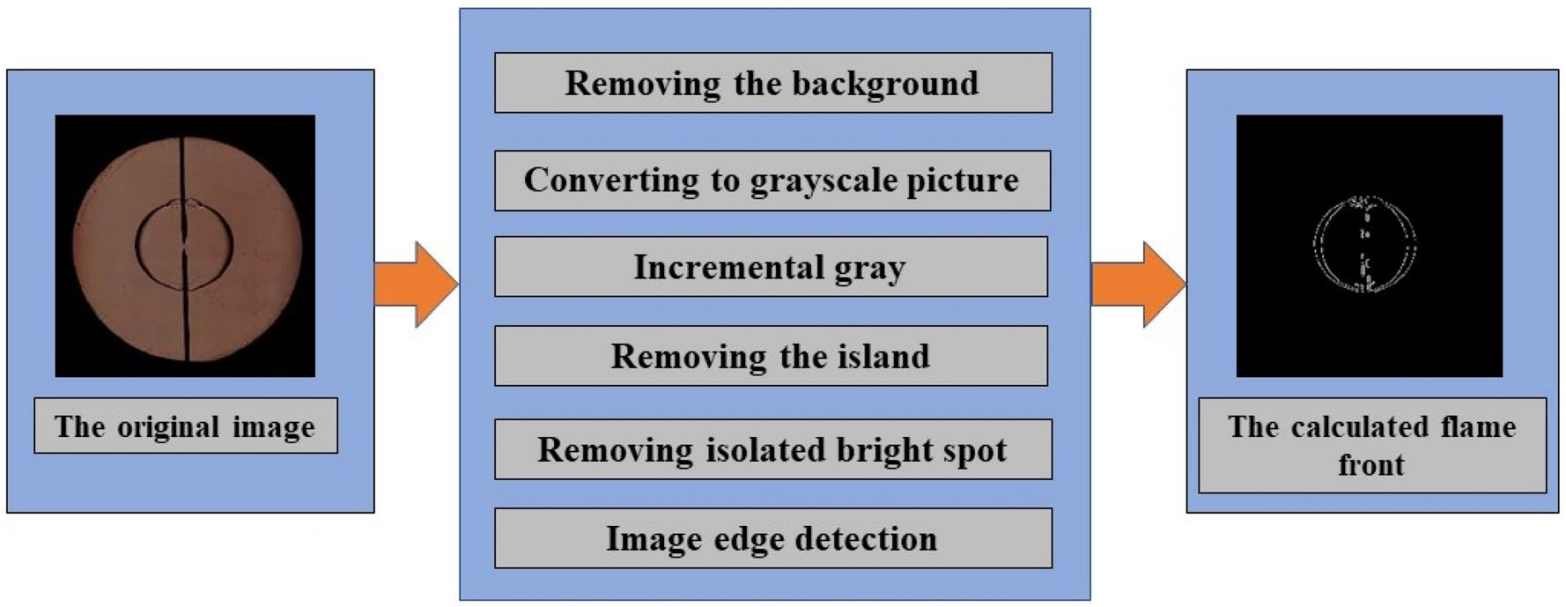
Flame image processing by MATLAB.

The flame image processing is shown in Fig. 4. A program using MATLAB code that is independently developed by the present research group is employed to process the Schlieren images through six steps: removing the background, transforming it into a gray image, increasing the gray level, removing the island, removing isolated highlights, and detecting image edges.

## Calculation methods and results discussion

### Calculation method

**Figure 5:**
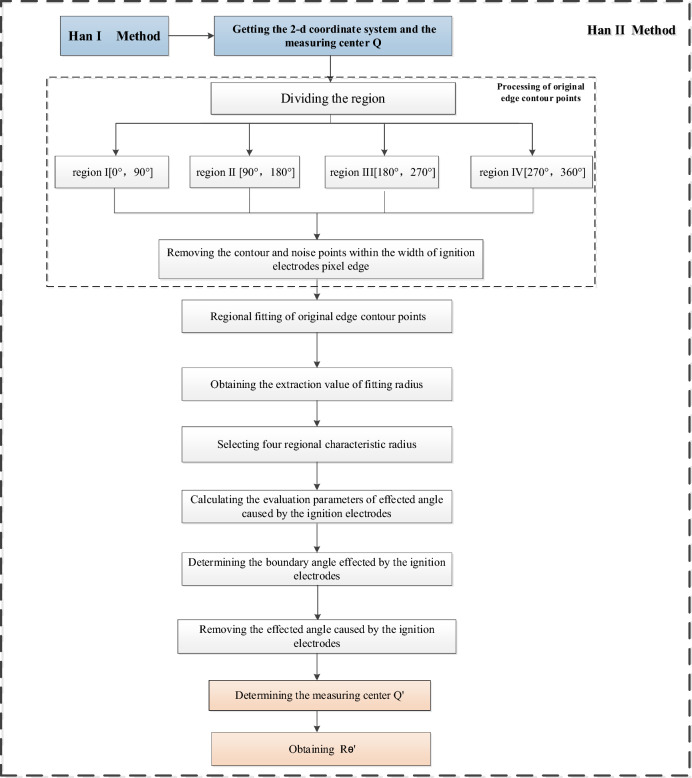
Frame diagram of the Han II method.

The two-dimensional coordinate system and measuring center Q of the Schlieren images are obtained based on the Han I method. The Schlieren images are divided into four regions. Then, the fitted edge contour points and extracted value of the fitting radius (${r}_{\theta }$) are obtained after removing the ignition electrodes and noise points of the images. The characteristic radius (${r}_{k}$) is the extracted value of the fitting radius in the direction toward the ignition electrodes in each of the four areas. The affected angle caused by the ignition electrodes (${\theta }_{b}$) is determined based on the values of the evaluation parameters of the affected angle caused by the ignition electrodes (${\Delta r}_{max}$). The above method for calculating the flame radius with greater accuracy by removing the ignition electrode effects supplements and improves the Han I method. In this paper, the optimized method is named the Han II method. The flame edge contour points are obtained using the output results of the method, measurement center Q', and the flame radius after removal of the ignition electrode effects (${R}_{\theta }^{\prime}$) are obtained by removing the angle of the ignition electrode effects, as shown in Fig. 5.

#### Preprocessing of original edge contour points

**Figure 6:**
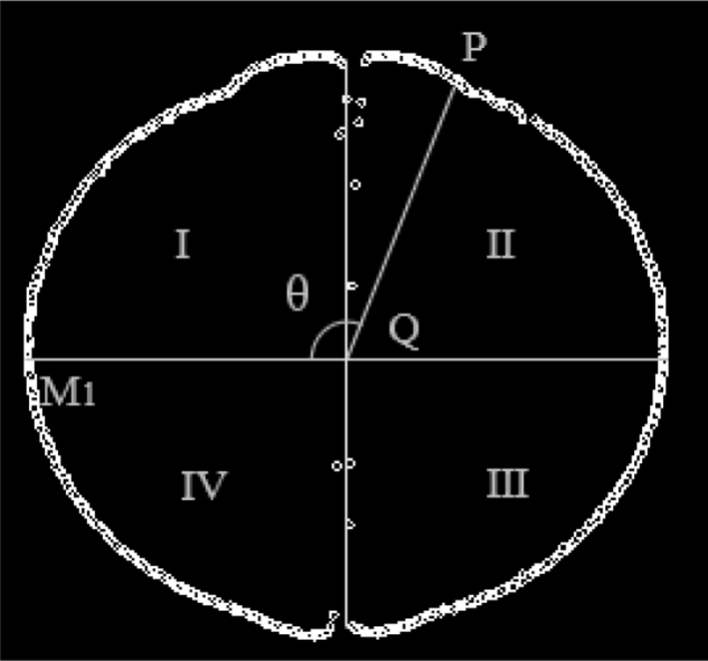
Dividing the regions.

In Fig. 6, a two-dimensional coordinate system is established based on measurement center point Q, which is determined by the Han I method^9^. The Schlieren images are divided into four regions using the horizontal and vertical coordinate system. The corresponding angle regions are defined as region I (0°–90°), region II (90°–180°), region III (180°–270°), and region IV (270°–360°), respectively. Passing through measurement center point Q in the direction of the ignition electrode connection is taken as the vertical axis, and the straight line perpendicular to the vertical axis is the horizontal axis. The intersection point between one side of the horizontal axis and the edge of the flame front surface is M_1_, which is the starting point. Then, QM_1_ is a fixed edge of the measuring angle in this coordinate system. In determining any flame front surface edge contour point P in the clockwise direction, angle M_1_QP is measurement angle $\theta$ ($\theta \in [0^\circ , 360^\circ ]$) in this coordinate system.

**Figure 7:**
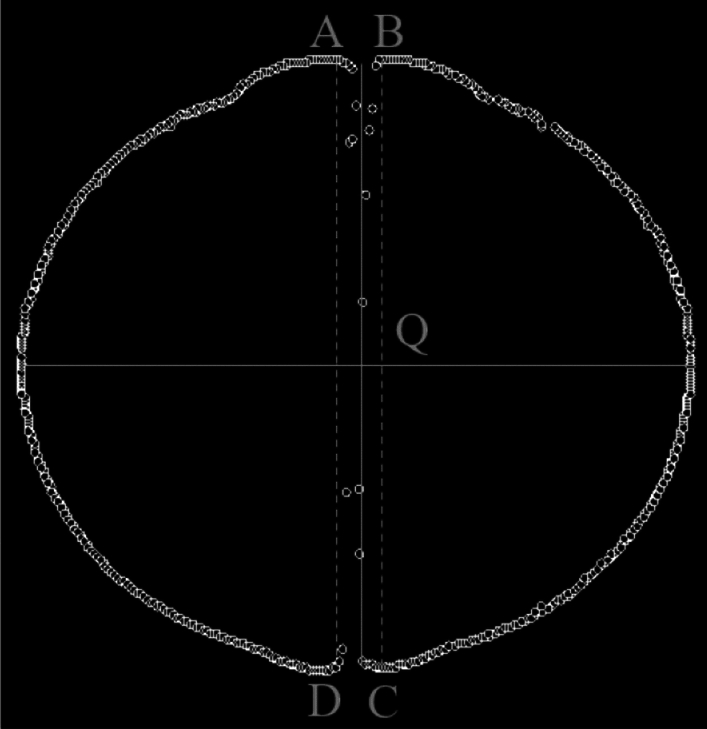
Removing the contour and noise points within the width of the ignition electrode pixel edge.

In the process of brightening gray-scale images through processing, numerous contour points and nearby noise points will be generated near the ignition electrodes. This will greatly affect the recognition of contour points by the MATLAB code. It is thus necessary to remove the contour points and nearby noise points of the ignition electrodes. The intersection points between the outline of ignition electrodes and the flame edge are A, D and B, C in Fig. 7. The ignition electrode pixel edge is defined by the two straight lines that pass-through points A, D and B, C, respectively. The contour and noise points within the width of the ignition electrode pixel edge must be removed.

#### Regional fitting of edge contour points

**Figure 8:**
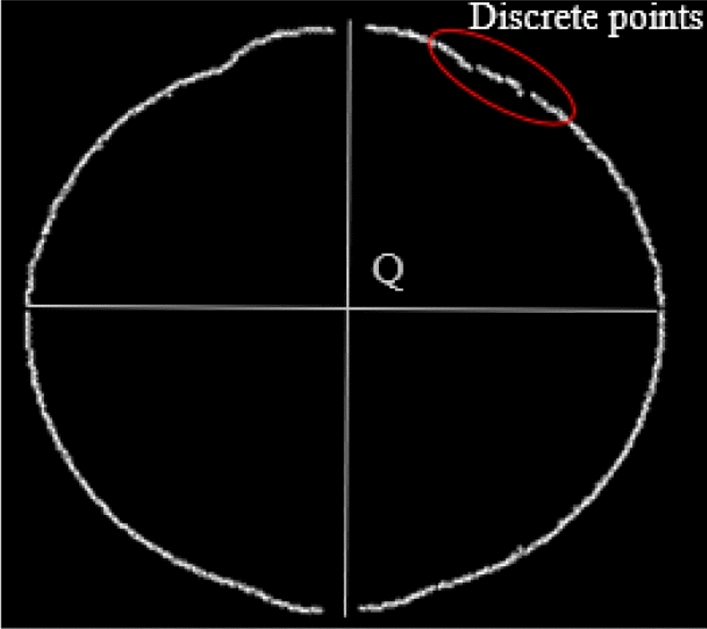
Discrete points of edge contour.

The corresponding random errors are generated since the original edge contour points are discrete points with an uneven distribution and randomness in Fig. 8. This makes it difficult to identify the ignition electrode effect boundary. To effectively eliminate random errors, the original contour points must be fitted because the correlation coefficients of the fitting above the ninth order are very close. Since the polynomial fitting coefficients are almost equal when the polynomial order is greater than 9, the least square method is used to fit the original contour points with nine-order polynomial.

**Figure 9:**
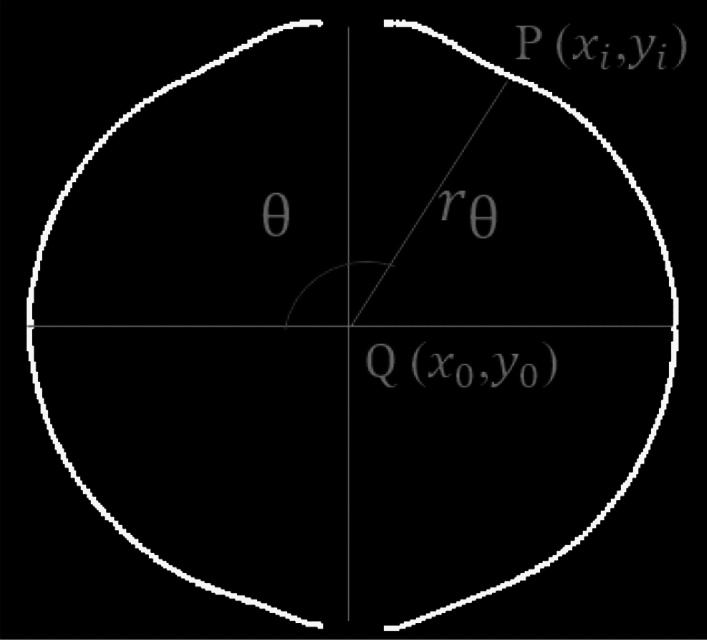
Calculating the extracted value of fitting radius (${r}_{\theta }$).

After obtaining the fitting edge contour points, a two-dimensional coordinate system is established using Schlieren images with measurement center point Q as the center of the circle, as determined by the Han I method. As shown in Fig. 9, the coordinates of point Q and point P in the coordinate system are defined as (${x}_{0}$,${y}_{0}$) and (${x}_{i}$,${y}_{i}$), respectively. In region I, it can be observed that the relation between line PQ and the corresponding angle θ is

1 $$\theta =arctan[({y}_{0}-{y}_{i})/({x}_{0}-{x}_{i})]$$

In region II and III, the relation between line segment PQ and corresponding angle θ is

2 $$\theta =arctan[({y}_{0}-{y}_{i})/({x}_{0}-{x}_{i})]+180$$

In region IV, the relation between line segment PQ and corresponding angle θ is

3 $$\theta =arctan[({y}_{0}-{y}_{i})/({x}_{0}-{x}_{i})] +360$$

The pixel difference between two points of PQ can be obtained by the circle radius function formula:

4 $${L}_{PQ} =\sqrt{({{x}_{0}-{x}_{i})}^{2}+{({y}_{0}-{y}_{i})}^{2}}$$

The extracted value of the fitting flame radius (${r}_{\theta }$) can be calculated from calibration ratio K^21^, $\theta \in [0^\circ , 84^\circ ] \cup [96^\circ , 264^\circ ] \cup [276^\circ , 360^\circ ]$. The equation is as follows:

5 $${r}_{\theta }={L}_{PQ}*K$$

Here, calibration ratio $K=S/{S}_{0}$, where *S* is the actual size of the scale, and ${S}_{0}$ is the pixel difference corresponding to the actual size of the scale in the Schlieren images.

**Figure 10:**
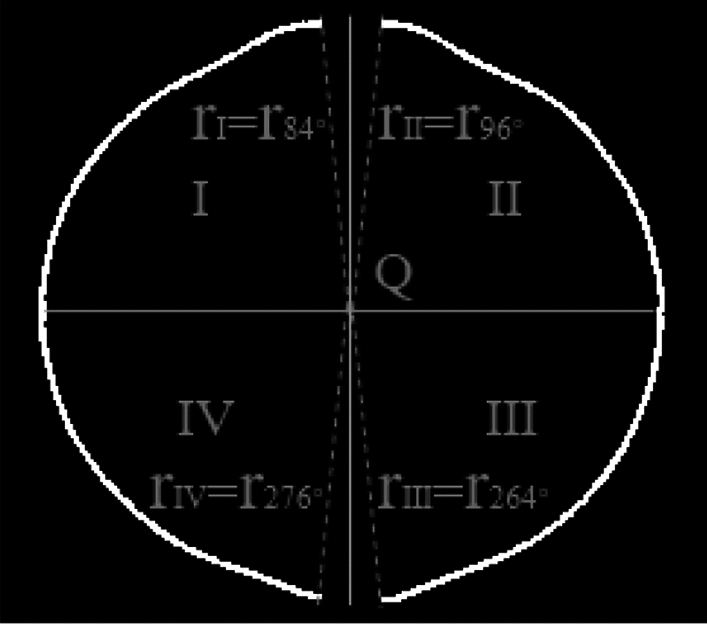
Definition of characteristic radius.

The extracted value of the fitting radius (${r}_{\theta }$), $\theta \in [0^\circ , 84^\circ ]\cup [96^\circ , 264^\circ ]\cup [276^\circ , 360^\circ ]$ is obtained. The extracted radius value ${r}_{84^\circ }$ in region I is defined as the characteristic radius ${{\text{r}}}_{{\text{I}}}$, the extracted radius value ${r}_{96^\circ }$ in region II is defined as the characteristic radius ${{\text{r}}}_{{\text{II}}}$, the extracted radius value ${r}_{264^\circ }$ in region III is defined as the characteristic radius ${{\text{r}}}_{{\text{III}}}$, the extracted radius value ${r}_{276^\circ }$ in region IV is defined as the characteristic radius ${{\text{r}}}_{{\text{IV}}}$ in Fig. 10.

#### Removing the angle affected by the ignition electrodes

**Figure 11:**
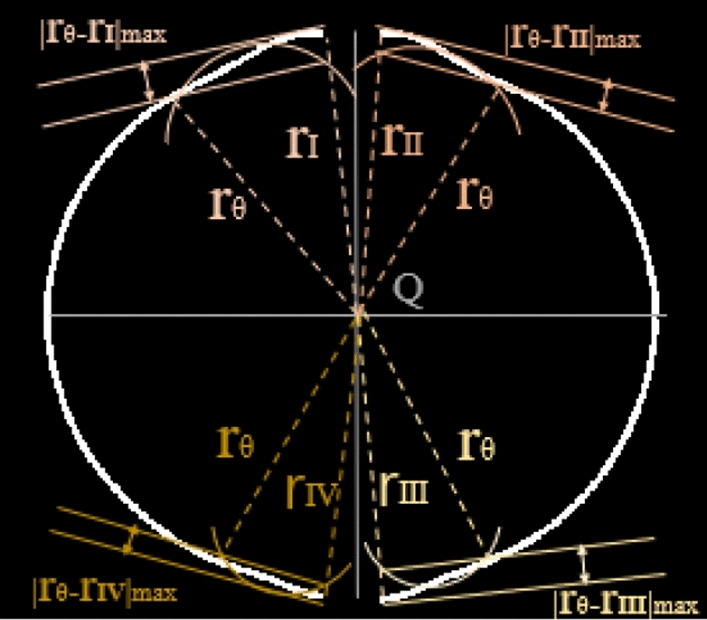
Determining the parameters.

The effects of the ignition electrodes on the flame edge contour are manifested as the mutation region, which includes the convex and concave shapes of the flame. As shown in Fig. 11, the maximum difference between the extracted value of the fitting radius (${r}_{\theta }$) and the characteristic radius (${r}_{k}$) can properly reflect the effects of the mutation region caused by the ignition electrodes on the flame propagation radius. Thus, the evaluation parameters of the angle affected by the ignition electrodes ($\Delta {r}_{max}$) for the four regions are defined as

6 $$\Delta {r}_{max}={|{r}_{\theta }-{r}_{k}|}_{max} (k=I,II,III,IV)$$

**Figure 12:**
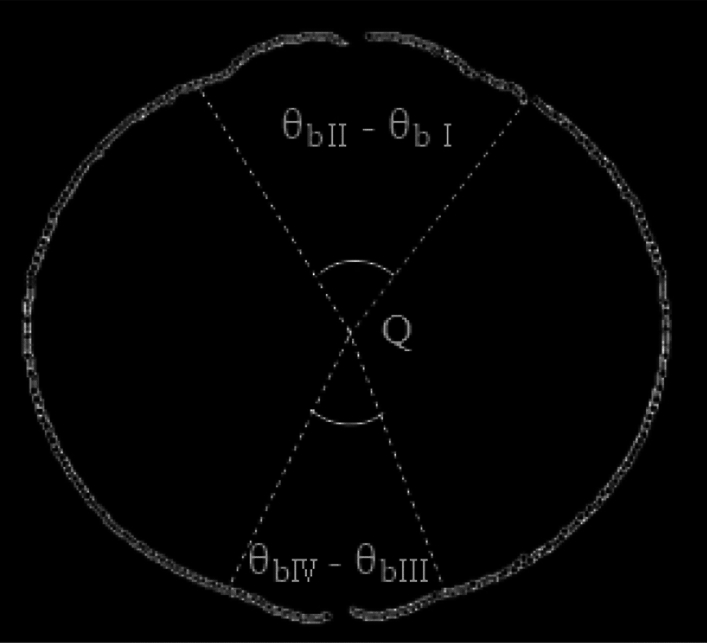
Removing the angle affected by the ignition electrodes.

In Eq. (6), extracted radius value ${r}_{\theta }$ is calculated first from the flame radius near the direction of the ignition electrode connection. The abrupt boundary of the mutation region caused by the ignition electrodes can be obtained from Eq. (6). Thus, the boundary angle (${\theta }_{bk}$) of each of the four regions is obtained from $\Delta {r}_{max}$ in Fig. 12. It is calculated by

7 $${\theta }_{b}=({\theta }_{bII}-{\theta }_{bI})+({\theta }_{bIV}-{\theta }_{bIII})$$

**Figure 13:**
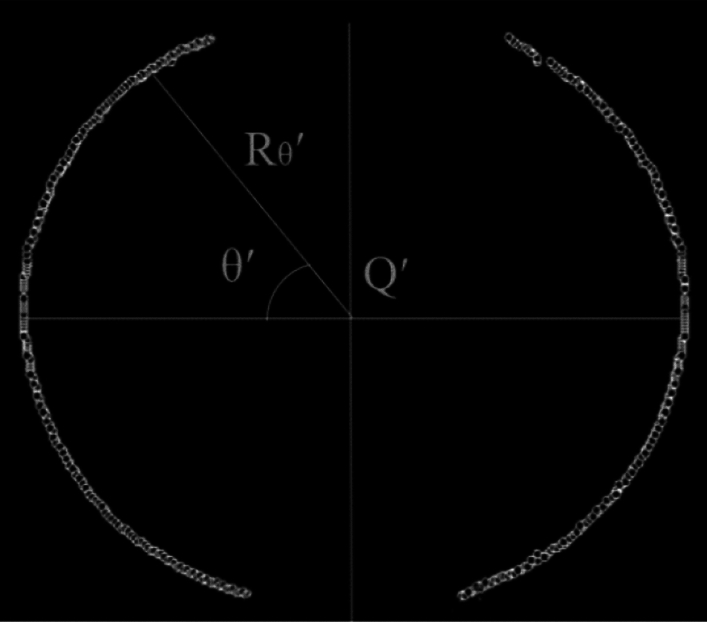
Re-determining the measurement center.

The new measurement center Q′ is determined again according to the Han I method^9^ after removing the angle affected by the ignition electrodes in Fig. 13. Here, $\theta ^{\prime}$ is defined as the measurement angle based on measurement center Q' in the two-dimensional coordinate system, and ${R}_{\theta }$′ is flame radius after removal of ignition electrode effects corresponding to angle $\theta ^{\prime}$. Thus, the mean radius calculated through the Han II method (${Ra}_{2}$) can be obtained.

### Evaluation index

To evaluate the optimization effect of the Han II method compared with the Han I method, the optimization rate of the mean radius ($\Delta Ra /{Ra}_{1}$) is used to describe them. The standard deviation optimization rate of the extracted radius value ($\Delta \sigma /{\sigma }_{1}$) and the range optimization rate of the extracted radius value ($\Delta R/{R}_{1}$) are used to describe the distribution rule. The difference of $\Delta Ra /{Ra}_{1}$ with time is described by the optimization rate of the flame mean radius per unit angle (${R}_{d}$). The difference of $\Delta \sigma /{\sigma }_{1}$ with time is described by the standard deviation optimization rate of the flame radius per unit angle (${\sigma }_{d}$). The specific evaluation of characteristic parameters is defined as follows:

1. Optimization rate of mean radius ($\Delta Ra /{Ra}_{1}$):
  8 $$\Delta Ra /{Ra}_{1}=({Ra}_{2}-{Ra}_{1})/{Ra}_{1}$$
  where ${Ra}_{1}$ and ${Ra}_{2}$ are the mean radius calculated by the Han I method and Han II method, respectively. This evaluation index is used to describe the optimization rate of the flame propagation radius.
  9 $${Ra}_{1}=\sum_{\theta =1}^{n}{R}_{\theta }/n$$
  10 $${Ra}_{2}= \sum_{\theta =1}^{n}{R}_{\theta }^{\prime}/n$$
  where ${R}_{\theta }$ and ${R}_{\theta }^{\prime}$ are the extracted radius values calculated by the Han I method and Han II method, respectively. $n$ is the total number of radius extracted values.
2. Optimization rate of the flame mean radius per unit angle (${R}_{d}$):
  11 $${R}_{d}=\Delta Ra/{\theta }_{b}$$
  where $\Delta Ra$ is the difference of the mean radius calculated by the Han I method and Han II method, respectively, and ${\theta }_{b}$ is the angle affected by the ignition electrodes. This evaluation index is used to describe the difference of the mean radius per unit angle.
3. Standard deviation optimization rate of the extracted radius value ($\Delta \sigma /{\sigma }_{1}$):
  12 $$\Delta \sigma /{\sigma }_{1}=({\sigma }_{1}-{\sigma }_{2})/{\sigma }_{1}$$
  where ${\sigma }_{1 }$ and ${\sigma }_{2}$ are the standard deviation of extracted radius values calculated by the Han I method and Han II method, respectively. This evaluation index quantifies the improvement of the non-uniform flame propagation state.
  Standard deviation of the extracted radius value ($\sigma$):
  13 $${\sigma }_{1}=\sqrt{{\sum_{\theta =1}^{n}({R}_{\theta }-{Ra}_{1})}^{2}/n}$$
  14 $${\sigma }_{2}=\sqrt{{\sum_{\theta =1}^{n}({{R}_{\theta }}^{\mathrm{^{\prime}}}-{Ra}_{2})}^{2}/n}$$
4. Standard deviation optimization rate of the flame radius per unit angle (${\sigma }_{d}$):
  15 $${\sigma }_{d}=\Delta \sigma /{\theta }_{b}$$
  where $\Delta\upsigma$ is the standard deviation of the extracted radius value calculated by the Han I method and Han II method, respectively, and ${\theta }_{b}$ is the angle affected by the ignition electrodes. This evaluation index is used to describe the standard deviation per unit angle.
5. Range optimization rate of the extracted radius value ($\Delta R/{R}_{1}$):
  16 $$\Delta R/{R}_{1}=({R}_{1}-{R}_{2})/{R}_{1}$$
  where ${R}_{1}$ and ${R}_{2}$ are the range of the extracted radius values calculated by the Han I method and Han II method, respectively. This evaluation index is used to describe the optimization effects when the flame presents both convex and concave distributions.

Range calculated through the Han I method (${R}_{1}$):

17 $${R}_{1}={R}_{\theta max}-{R}_{\theta min}$$

Range calculated through the Han II method (${R}_{2}$):

18 $${R}_{2}={{{R}_{\theta }}^{\mathrm{^{\prime}}}}_{max}-{{{R}_{\theta }}^{\mathrm{^{\prime}}}}_{min}$$

where ${R}_{\theta max}\mathrm{ and}$ ${R}_{\theta min}$ are the maximum and minimum extracted radius values calculated by the Han I method respectively. ${{{R}_{\theta }}^{\mathrm{^{\prime}}}}_{max}$ and ${{{R}_{\theta }}^{\mathrm{^{\prime}}}}_{min}$ are the maximum and minimum extracted radius values calculated by the Han II method, respectively.

### Adaptability analysis

**Figure 14:**
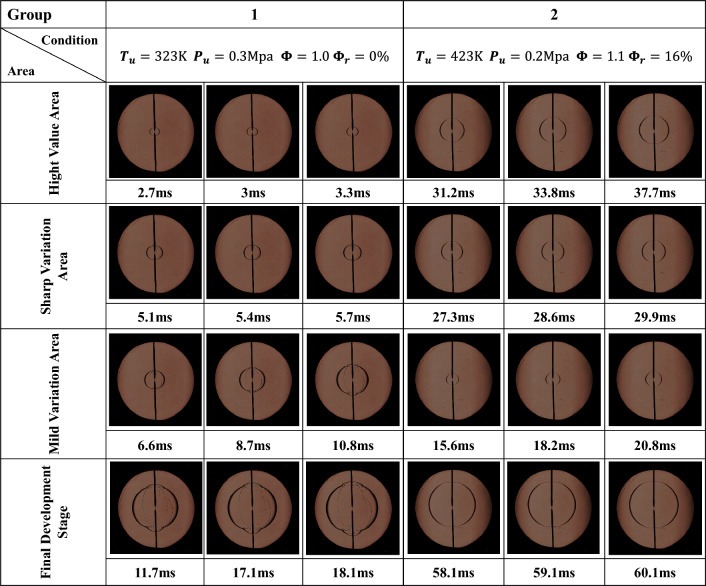
Schlieren images of the two typical working conditions.

Two typical working conditions in which the flame presents a convex or concave distribution are selected to conduct the adaptability analysis of the Han II method, as well as to investigate the affected rule of ignition electrodes on the flame propagation radius. Figure 14 shows the Schlieren images of the two typical working conditions.

The ratio of the angle affected by ignition electrodes $P$ is defined as follows:

19 $$P=\left({\theta }_{b}/360\right)\times 100\%$$

where ${\theta }_{b}$ is the angle affected by the ignition electrodes.

**Figure 15:**
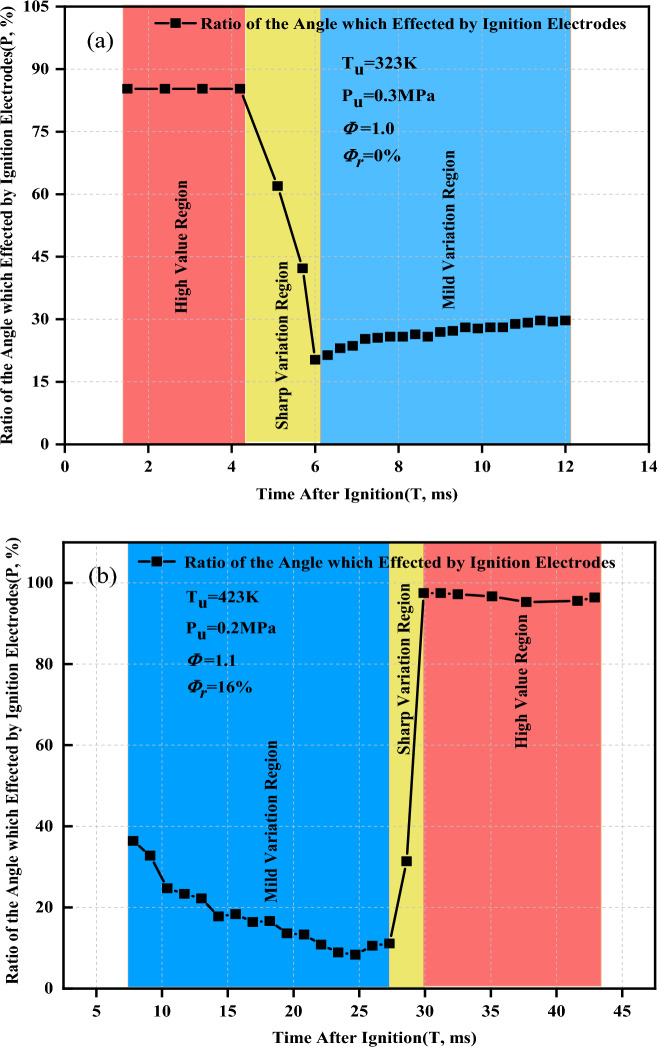
Ratio of the angle affected by the ignition electrodes of two typical working conditions. (**a**) Schlieren images of the flame presenting a convex distribution; (**b**) Schlieren images of the flame presenting a concave distribution.

Figure 15 displays the ratio of the angle affected by the ignition electrodes of the two typical working conditions. The flame propagation radius is 6–25 mm. The flame schlieren images with flame development time between 1.5–12 ms and 7.8–42.9 ms under convex or concave flame distributions were selected for analysis. Figure 15a shows the ratio of the angle affected by the ignition electrodes when the flame presented a convex distribution. It can be observed that the maximum ratio of the angle is 84–85% when the flame development time is 1.5 to 4.2 ms. As the flame continues to spread, the ratio of the angle decreases dramatically from 85 to 21% when the flame development time is 4.2 to 6.0 ms. However, the ratio eventually begins to slightly increase again from 21 to 30% when the flame development time is 4.2 to 6.0 ms. The ratio of the angle can be divided into three regions including a high value region, sharp variation region, and mild variation region for the flame that presents a convex distribution.

With in-depth analysis of the Schlieren images showing the flame presenting a convex distribution, the boundary of the flame is affected by the ignition electrodes may have been misjudged for the different flame propagation stages, resulting in a large ratio of the angle affected by the ignition electrodes. The flame development time is 1.5 to 4.2 ms, and the flame front remains smooth for the high-value region, which may lead to a misjudgment of the boundary in Fig. 15a. Therefore, the ratio of the angle affected by the ignition electrodes in the high-value region is not reliable. For the sharp-variation region, the flame development time is 4.2 to 6.0 ms. The flame front surface near the ignition electrodes gradually appears slightly convex, which causes a decrease of the ratio of the angle affected by the ignition electrodes. The error of the boundary also gradually decreases. However, owing to the small value of ${\Delta r}_{max}$ (1.52 to 1.64 mm), the ratio of the angle affected by the ignition electrodes in this region is also not reliable. For the mild-variation region, the flame development time is 1.64 to 2.65 ms, and the slightly convex shape of the flame front surface near the ignition electrodes develops into an obviously convex shape. Here, ${\Delta r}_{max}$ is increased from 1.64 to 2.65 mm, which is sufficient to identify the boundary. Thus, the ratio of the angle affected by the ignition electrodes in the mild-variation region can be considered highly reliable. It can be concluded that the ratio of the angle affected by the ignition electrodes is only applicable to the mild-variation region when the flame presents a convex distribution.

Figure 15b shows the ratio of the angle affected by the ignition electrodes when the flame presents a concave distribution. The ratio of the angle can be divided into three regions include a mild-variation region, sharp-variation region, and high-variation region. The ratio of the angle affected by the ignition electrodes shows a relatively small reduction from 37 to 11% when the flame development time is 7.8 to 27.3 ms. This region is defined as a mild-variation region. As the flame continues to spread, the ratio of the angle increases dramatically from 11 to 98% when the flame development time is 27.3 to 29.9 ms. This region of the sudden change is defined as a sharp-variation region. The ratio of the angle affected by the ignition electrodes eventually increases to more than 95% when the flame development time is 29.9 to 42.9 ms. Hence, this region is defined as a high-value region. It can be concluded that the ratio of the angle is only applicable to the mild-variation region when the flame presents a concave distribution.

### Evaluation of calculation method

To systematically evaluate the accuracy of the flame radius after the removal of the ignition electrode effects in the convex distribution, the above evaluation of characteristic parameters are used to describe the flame propagation radius and the difference of optimization.

**Figure 16:**
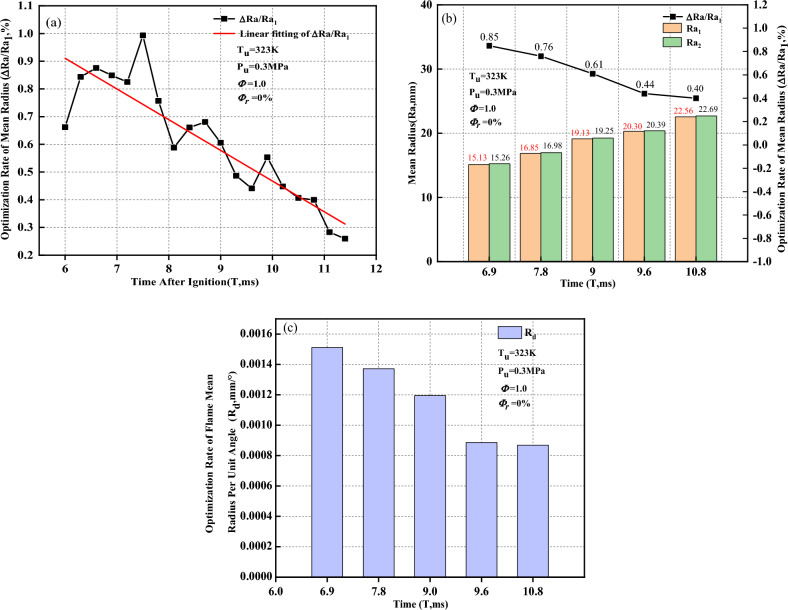
Optimization rate of the mean radius when the flame presents a convex distribution. (**a**) Optimization rate of the mean radius; (**b**) Mean radius and the optimization rate of the mean radius; (**c**) Optimization rate of the flame mean radius per unit angle.

As the flame propagates, the optimization rate of the mean radius ($\Delta Ra/{\Delta Ra}_{1}$) tends to linearly decrease in Fig. 16a. The Schlieren images at five different moments within the flame time of 6 to 12 ms are selected for analysis. Figure 16b shows the mean radius $(Ra)$ and the optimization rate of the mean radius versus time when the flame presents a convex distribution. After removal of the ignition electrode effects, the mean radius increases, and the increment rate is between 0.4 and 0.85%. As the flame propagates, the optimization rate of the mean radius gradually decreases. The optimization rate of the flame mean radius per unit angle (${R}_{d}$) gradually decreases, which is why $\Delta Ra/{\Delta Ra}_{1}$ decreases as the flame propagates in Fig. 16c.

**Figure 17:**
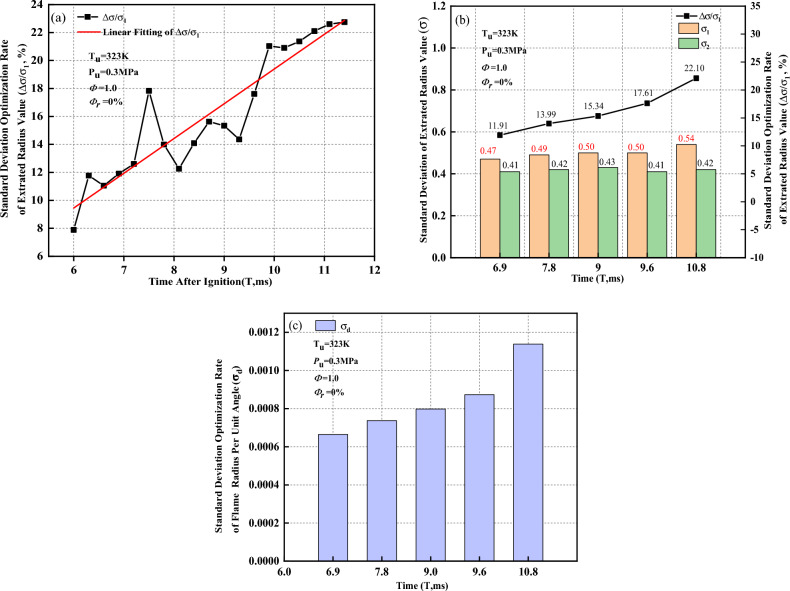
Optimization rate of standard deviation when flame presents convex distribution. (**a**) Standard deviation optimization rate of extracted radius value; (**b**) Standard deviation of extracted radius value and standard deviation optimization rate of extracted radius value; (**c**) Standard deviation optimization rate of flame radius per unit angle.

The standard deviation optimization rate of the extracted radius value (Δσ/${\sigma }_{1}$) tends to linearly increase as the flame propagates in Fig. 17a. In this study, Schlieren images at five different moments within the flame time of 6 to 12 ms are selected for analysis. Figure 17b shows the standard deviation of extracted radius value $\sigma$ and the standard deviation optimization rate of the extracted radius value versus time when the flame presents a convex distribution. After removal of the ignition electrode effects, the standard deviation decreases, which implies that the inhomogeneity of the flame-radius extracted values decreases, and the reduced rate is between 11.91 and 22.1%. As the flame propagates, the standard deviation optimization rate of the extracted radius value gradually increases.

In Fig. 17c, the standard deviation optimization rate of the flame radius per unit angle (${\sigma }_{d}$) gradually increases as the flame propagates, which why $\Delta \sigma /{\sigma }_{1}$ increases. This implies that the extracted radius values calculated by the Han II method are closer to the actual flame propagation radius. The convex distribution near the ignition electrodes is caused by the irregular shape of the initial flame core. However, the resistance caused by the flame-retarding effect near the ignition electrode surface inhibits the convex distribution near the ignition electrodes. Meanwhile, the stretching effect during flame development also inhibits a convex distribution near the ignition electrodes. With the flame development, the self-accelerating phenomenon aggravates the convex tendency near the ignition electrodes to a certain extent. In the process of the combined effects of those four factors, the self-accelerating effect of the flame is dominant and its effect is greater than the ignition electrode surface-retarding effect and the flame-stretching effect. The flame near the ignition electrodes presents a convex distribution and tends to change more intensely. The specific mechanism of the influence of those four factors on the flame shape near the ignition electrodes needs further study.

**Figure 18:**
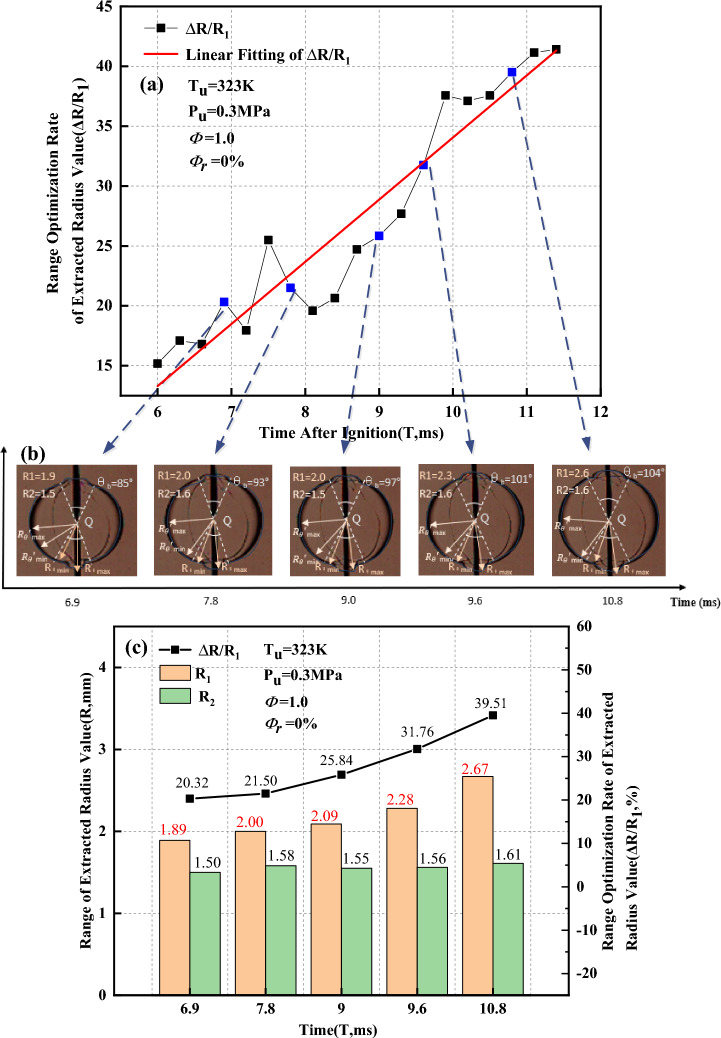
Range of the extracted radius value when the flame presents a convex distribution.

In Fig. 18a, the range optimization rate of the extracted radius value ($\Delta R/{R}_{1}$) tends to linearly increase as the flame propagates. Thus, Schlieren images at five different moments within the flame time of 6 to 12 ms are selected for analysis. Figure 18b shows the distribution of the maximum and minimum extracted radius values respectively calculated by the Han I method and Han II method when the flame presents a convex distribution. It can be observed that the maximum and minimum extracted radius values calculated by the Han I method are distributed within the angle affected by the ignition electrodes. However, the maximum and minimum extracted radius values calculated by the Han II method are distributed outside the angle affected by the ignition electrodes. Figure 18c shows the range of the extracted radius value ($R$) and the range optimization rate of the extracted radius value versus time when the flame presents a convex distribution. After removal of the ignition electrode effects, the range of the extracted radius value decreases, and the reduced rate of $R$ is between 20.32 and 39.51%. As the flame propagates, the range optimization rate of the extracted radius value ($\Delta R/{R}_{1}$) gradually increases. This implies that the Han II method can effectively remove the ignition electrode effects.

**Figure 19:**
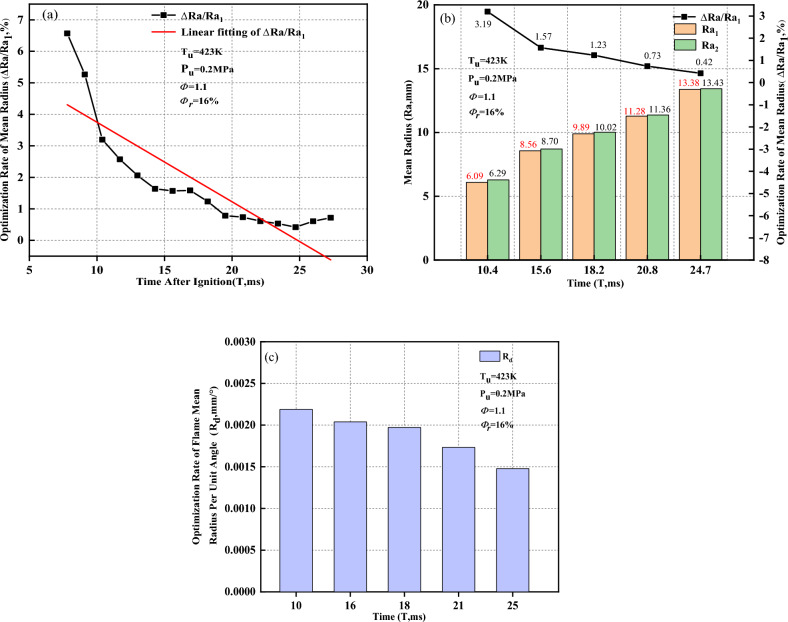
Optimization rate of the mean radius when the flame presents a concave distribution. (**a**) Optimization rate of mean radius; (**b**) Mean radius and optimization rate of mean radius; (**c**) Optimization rate of flame mean radius per unit angle.

As shown in Fig. 19a, as the flame propagates, the optimization rate of the mean radius ($\Delta Ra/{\Delta Ra}_{1}$) tends to linearly decrease. Thus, Schlieren images at five different moments within the flame time of 7.8 to 27.3 ms are selected for analysis. Figure 19b shows the mean radius $(Ra)$ and optimization rate of the mean radius versus time when the flame presents a concave distribution. After removal of the ignition electrode effects, the mean radius increases, and the increment rate is between 0.42 and 3.19%. As the flame propagates, the optimization rate of the mean radius gradually decreases. As shown in Fig. 19c, the optimization rate of the flame mean radius per unit angle (${R}_{d}$) gradually decreases, which is why $\Delta Ra/{\Delta Ra}_{1}$ decreases as the flame propagates.

**Figure 20:**
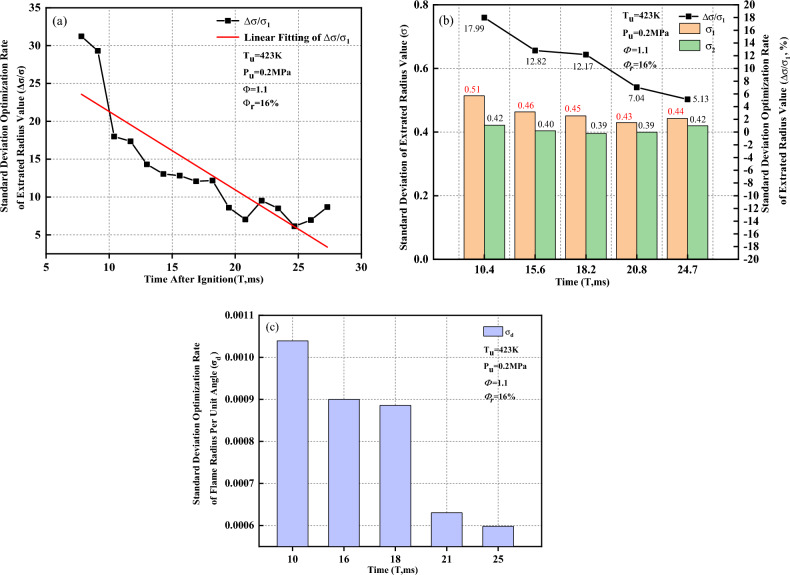
Optimization rate of the standard deviation when the flame presents a concave distribution. (**a**) Standard deviation optimization rate of extracted radius value; (**b**) Standard deviation of extracted radius value and standard deviation optimization rate of extracted radius value; (**c**) Standard deviation optimization rate of flame radius per unit angle.

As shown in Fig. 20a, the standard deviation optimization rate of the extracted radius value (Δσ/${\sigma }_{1}$) tends to linearly decrease as the flame propagates. In this study, Schlieren images at five different moments within the flame time of 7.8 to 27.3 ms are selected for analysis. Figure 20b shows the standard deviation of the extracted radius value $(\sigma$) and the standard deviation optimization rate of the extracted radius value versus time when the flame presents a concave distribution. After removal of the ignition electrode effects, the standard deviation decreases, which implies that the inhomogeneity of the flame-radius extracted values decreases, and the reduce rate is between 5.13 and 17.99%. As the flame propagates, the standard deviation optimization rate of the extracted radius value gradually decreases.

As shown in Fig. 20c, the standard deviation optimization rate of the flame radius per unit angle (${\sigma }_{d}$) gradually decreases as the flame propagates, which why $\Delta \sigma /{\sigma }_{1}$ decreases. This implies that the extracted radius value calculated by the Han II method is closer to the actual flame propagation radius; however, it is slightly different from the variation trends that occur when the flame presents a convex distribution. The convex distribution near the ignition electrodes is caused by the irregular shape of the initial flame core. However, the resistance caused by the flame-retarding effect near the ignition electrode surface inhibits the convex distribution near the ignition electrodes. Meanwhile, the stretching effect during flame development also inhibits the convex distribution near the ignition electrodes. With the flame development, the self-accelerating phenomenon aggravates the convex tendency near the ignition electrodes to a certain extent. In the process of the combined effects of those four factors, the ignition electrode surface retarding and flame stretching are dominant. The convex tendency of the initial flame fire core is gradually inhibited until it reverses. The flame near the ignition electrodes displays a concave distribution, and it tends to slow under this working condition with the flame development. However, the specific mechanism of those four factors on the flame shape near the ignition electrodes needs further study.

**Figure 21:**
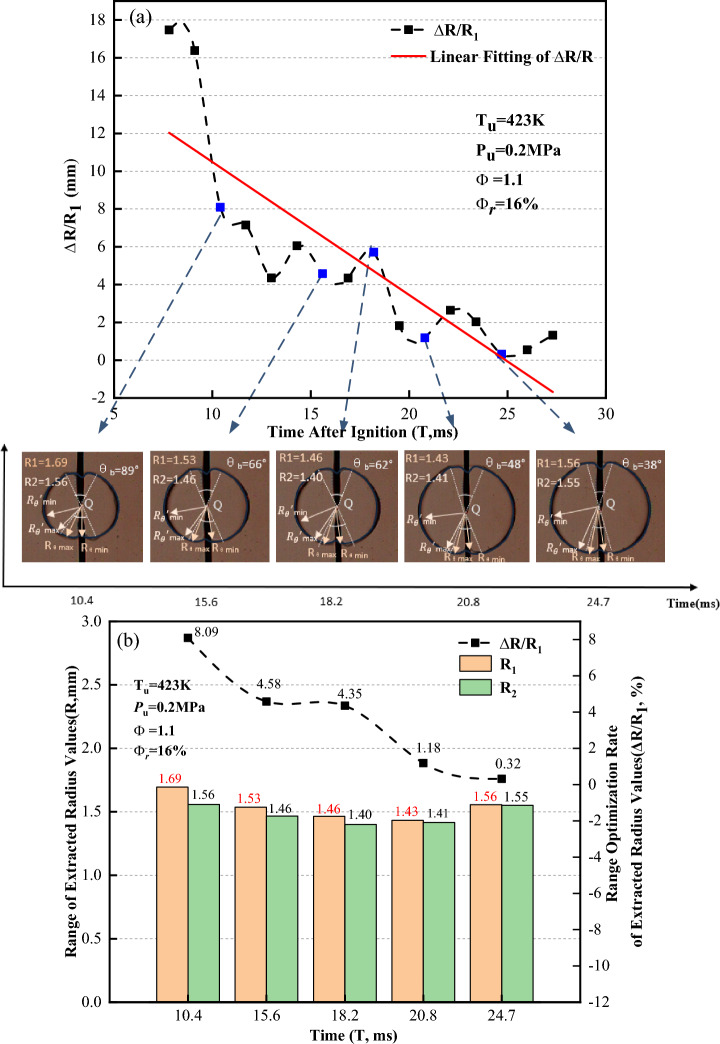
Range of extracted radius values when flame presents a concave distribution.

In Fig. 21a, the range optimization rate of the extracted radius value ($\Delta R/{R}_{1}$) tends to linearly decrease as the flame propagates. Schlieren images at five different moments within the flame time of 7.8 to 27.3 ms are selected for analysis. Figure 21b shows the distribution of the maximum and minimum extracted radius values respectively calculated by the Han I method and Han II method when the flame presents a concave distribution. The maximum and minimum extracted radius values calculated by the Han I method are distributed within the angle affected by the ignition electrodes; nevertheless, the maximum and minimum extracted radius values calculated by the Han II method are distributed outside the angle affected by the ignition electrodes. Figure 21c shows the range of the extracted radius value ($R$) and range optimization rate of the extracted radius value ($\Delta R/{R}_{1}$) versus time when the flame presents a concave distribution. After removal of the ignition electrode effects, the range of the extracted radius value ($R$) decreases, and the reduced rate of $R$ is between 0.32 and 8.09%. As the flame propagates, the range optimization rate of the extracted radius value ($\Delta R/{R}_{1}$) gradually decreases. This implies that the Han II method can effectively remove the ignition electrode effects, however, this trend is slightly different from the variation trends that occur when the flame presents a convex distribution. This is due to the different flame shapes resulting from the ignition electrode effects. The effect of the ignition electrodes is weakened as the flame propagates when presenting a concave distribution.

## Conclusions

In this paper, a flame-radius calculation method is proposed to remove the effects of ignition electrodes. The adaptability and optimization effects of this method are analyzed. The main conclusions are as follows:

1. The ratio of the angle affected by the ignition electrodes calculated by the Han II method showed three obvious divisions: a high-value area, sharp-variation area, and mild-variation area. The ratio of the angle affected by the ignition electrodes is only applicable to the mild-variation region when the flame presented respective convex and concave distributions.
2. In the case of the convex distribution, after removing the ignition electrode effects, the increment rate of the mean radius is 0.4–0.85%. The reduced rate of the standard deviation of the extracted radius value is 11.91–22.1%. The decreased rate of the range of the extracted radius value is 20.32–39.51%.
3. In the case of a concave distribution, after removing the ignition electrode effects, the increment rate of the mean radius is 0.42–3.19%. The reduced rate of the standard deviation of the extracted radius value is 5.13–17.99%. Moreover, the decreased rate of the range of the extracted radius value is 0.32–8.09%.

## Data Availability

The datasets used and/or analysed during the current study available from the corresponding author on reasonable request.

## List of symbols

${r}_{\theta }$: Extracted Value of Fitting Radius
$${r}_{k}$$: Characteristic Radius
${\Delta r}_{max}$: Evaluation Parameters of Affected Angle Due to Ignition Electrodes
$${\theta }_{bk}$$: Boundary Angle
${\theta }_{b}$: Affected Angle Due to Ignition Electrodes
$\theta$: Measurement Angle of Flame Radius
$${T}_{u}$$: Initial Temperature
$${P}_{u}$$: Initial Pressure
$$\Phi $$: Equivalence Ratio
${\Phi }_{r}$: Diluent Gas Fraction
CVCV: Constant Volume Combustion Vessel
${R}_{\theta }^{\prime}$: Flame Radius after Removal of Ignition Electrode Effects
*Ra*: Mean Radius
${Ra}_{1}$: Mean Radius Calculated through Han I Method
${Ra}_{2}$: Mean Radius Calculated through Han II Method
$R$: Range of Extracted Radius Value
${R}_{1}$: Range Calculated through Han I Method
${R}_{2}$: Range Calculated through Han II Method
$\sigma$: Standard Deviation of Extracted Radius Value
${\sigma }_{1}$: Standard Deviation Calculated through Han I Method
${\sigma }_{2}$: Standard Deviation Calculated through Han II Method
$P$: Ratio of Angle Affected by Ignition Electrodes
$\Delta Ra/\Delta {Ra}_{1}$: Optimization Rate of Mean Radius
${\Delta R/\Delta R}_{1}$: Range Optimization Rate of Extracted Radius Value
$\Delta \sigma /{\Delta \sigma }_{1}$: Standard Deviation Optimization Rate of Extracted Radius Value
${R}_{d}$: Optimization Rate of Flame Mean Radius Per Unit Angle
${\sigma }_{d}$: Standard Deviation Optimization Rate of Flame Radius Per Unit Angle

## References

[1] Huang Z, Zhang Y, Zeng K, Liu B, Wang Q, Jiang D (2006). Measurements of laminar burning velocities for natural gas–hydrogen–air mixtures. Combust Flame.

[2] Mohapatra S, Mohapatro MBR, Pasha AA (2022). Adaptability of different mechanisms and kinetic study of methane combustion in steam diluted environments. Sci. Rep..

[3] Cheng Q, Karimkashi S, Ahmad Z (2023). Hyperspectral image reconstruction from colored natural flame luminosity imaging in a tri-fuel optical engine. Sci. Rep..

[4] Wang Q, Xu X, Chang W (2022). Suppression of deflagration flame propagation of methane–air in tube by argon gas and explosion-eliminating chamber. Sci. Rep..

[5] Li, L. Research on determination of radii of spherical flames and computer realization. Ph.D. Thesis; Wuhan University of Technology (2014). 10.7666/d.D617237.

[6] Li H, Li G, Sun Z, Zhou Z, Li Y, Yuan Y (2016). Effect of dilution on laminar burning characteristics of H_2_/CO/CO_2_/air premixed flames with various hydrogen fractions. Exp. Therm. Fluid Sci..

[7] Chen TC, Chung K (2001). An efficient randomized algorithm for detecting circles. Comput. Vis. Image Undergr..

[8] Broustail G, Seers P, Halter F, Moreac G, Mounaim-Rousselle C (2011). Experimental determination of laminar burning velocity for butanol and ethanol iso-octane blends. Fuel.

[9] Han Z, Xiao B, Tian W, Li J, Yu W (2020). Study on difference and adaptability of calculation method of spherical flame radius. Fuel.

[10] Bouvet N, Chauveau C, Gökalp I, Halter F (2011). Experimental studies of the fundamental flame speeds of syngas (H_2_/CO)/air mixtures. Proc. Combust. Inst..

[11] Zuo Z, Pei Y, Qin J, Xu H, Lu L (2018). Laminar burning characteristics of premixed methane-dissociated methanol–air mixtures under lean burn conditions. Appl. Therm. Eng..

[12] Gu XJ, Haq MZ, Lawes M, Woolley R (2000). Laminar burning velocity and Markstein lengths of methane-air mixtures. Combust. Flame.

[13] Milton BE, Keck JC (1984). Laminar burning velocities in stoichiometric hydrogen and hydrogen hydrocarbon gas mixtures. Combust. Flame.

[14] Zhang X, Hou X, Wang Y, Zhang J (2019). Study on flame characteristics of low heat value gas. Energy Convers. Manag..

[15] DuttaRoy R, Chakravarthy SR, Sen AK (2020). Experimental investigation of flame propagation in a meso-combustor. Proc. Inst. Mech. Eng. A J. Power Energy.

[16] Gong X, Huo J, Ren Z, Law CK (2019). Extrapolation and DNS-mapping in determining laminar flame speeds of syngas/air mixtures. Combust. Flame.

[17] Tahtouh T, Halter F, Mounaïm-Rousselle C (2009). Measurement of laminar burning speeds and Markstein lengths using a novel methodology. Combust. Flame.

[18] Wu F, Jomaas G, Law CK (2013). An experimental investigation on self-acceleration of cellular spherical flames. Proc. Combust. Inst..

[19] Li G, Liang J, Zhang Z, Tian L, Cai Y, Tian L (2015). Experimental investigation on laminar burning velocities and markstein lengths of premixed methane–n-heptane–air mixtures. Energy Fuels.

[20] Wang X, Huang Z, Zhang Z, Xiang J, Wang X, Miao H, Jiang D (2009). Premixed combustion of spherically propagating methanol-air-nitrogen flames. Trans. CSICE.

[21] Han Z, Xiao B, Su Q, Wu X, Qian Y (2019). Study on accuracy of measuring propagation radius of constant volume combustion flame. Trans. CSICE.

[22] Mevel R, Lafosse F, Chaumeix N, Dupre G, Paillard CE (2009). Spherical expanding flames in H_2_–N_2_O-Ar mixtures: Flame speed measurements and kinetic modeling. Int. J. Hydrog. Energy.

[23] Varea E, Modica V, Vandel A, Renou B (2012). Measurement of laminar burning velocity and Markstein length relative to fresh gases using a new postprocessing procedure: Application to laminar spherical flames for methane, ethanol and isooctane/air mixtures. Combust. Flame.

[24] Xie Y, Wang J, Cai X, Huang Z (2016). Self-acceleration of cellular flames and laminar flame speed of syngas/air mixtures at elevated pressures. Int. J. Hydrog. Energy.

[25] Law CK, Sung C (2000). Structure, aerodynamics, and geometry of premixed flamelets. Prog. Energy Combust..

